# Mechanisms conferring bacterial cell wall variability and adaptivity

**DOI:** 10.1042/BST20230027

**Published:** 2024-09-26

**Authors:** Gabriel Torrens, Felipe Cava

**Affiliations:** Department of Molecular Biology and Laboratory for Molecular Infection Medicine Sweden, Umeå Centre for Microbial Research, SciLifeLab, Umeå University, Umeå, Sweden

**Keywords:** adaptation, antibiotic resistance, host-pathogen interaction, peptidoglycan

## Abstract

The bacterial cell wall, a sophisticated and dynamic structure predominantly composed of peptidoglycan (PG), plays a pivotal role in bacterial survival and adaptation. Bacteria actively modify their cell walls by editing PG components in response to environmental challenges. Diverse variations in peptide composition, cross-linking patterns, and glycan strand structures empower bacteria to resist antibiotics, evade host immune detection, and adapt to dynamic environments. This review comprehensively summarizes the most common modifications reported to date and their associated adaptive role and further highlights how regulation of PG synthesis and turnover provides resilience to cell lysis.

## Introduction

The bacterial cell wall is a complex and dynamic structure that surrounds the cell membrane of most bacteria. It provides shape, rigidity, and protection to cells, mainly by preventing osmotic rupture from high internal turgor pressure. Additionally, it plays crucial roles in cell division, nutrient uptake, and cell signaling [1]. This cell wall is mainly composed of peptidoglycan (PG), a polymer constituted of repeating units that form a three-dimensional network surrounding the cell. Structurally, this network features chains of the *N*-acetylglucosamine (NAG) and *N*-acetylmuramic acid (NAM) sugars connected via β-(1,4) glycosidic bonds. Additionally, these glycan strands are cross-linked via short peptides emerging from NAM. PG peptides are composed of L- and D-amino acids [2,3] and can vary in sequence and length between bacterial species. The most common amino acids found in the PG of Gram-negative bacteria are L-alanine (L-Ala), D-alanine (D-Ala), D-glutamic acid (D-Glu), and meso-diaminopimelic acid (mDAP) [4]. D-Ala and mDAP residues frequently engage in the cross-linking of adjacent glycan chains through specific enzymes known as DD-transpeptidases or Penicillin-binding proteins (PBPs), which serve as direct targets of β-lactam antibiotics. These enzymes facilitate the linkage of the fourth D-Ala from the donor stem peptide to the mDAP at the third position of the acceptor peptide. This 4-3 cross-link type is the main mechanism used by bacteria to cross-link their PG glycan strands. However, alternative cross-linking is catalyzed by LD-transpeptidases, enzymes that connect both mDAP residues from adjacent strands, resulting in a 3-3 cross-link type [4–6] or a less common and recently described LD-cross-linking between L-Ala and mDAP (type 1-3) in α- and β-proteobacteria [7]. Notably, LD-transpeptidases are often not efficiently inhibited by most β-lactams antibiotics, thus providing a bypass strategy against these cell wall-acting antibiotics [8].

**Figure 1.**
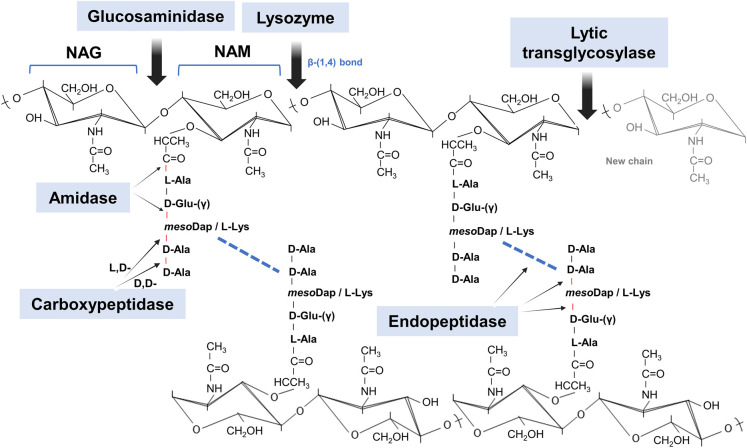
Chemical structure of peptidoglycan with enzymatic cleavage sites. Schematic representation of the archetypal structure of muropeptides consisting of NAG-NAM disaccharides attached to a peptide chain containing 2–5 typical amino acid residues. In this model, a DD-cross-linking (product of DD-transpeptidation by PBPs) is shown (blue dashed line). The main enzymatic degradative activities involved in PG adaptation are shown with their respective cleavage sites. NAG, *N*-acetyl-glucosamine; NAM, *N*-acetyl muramic acid; L-Ala, L-Alanine; D-Glu-(γ), D-glutamic acid; mesoDap or DAP, meso-diaminopimelic acid (Gram-negatives); L-Lys, L-lysine (Gram-positives); D-Ala, D-Alanine.

The mature PG is a dynamic structure that undergoes constant turnover to allow cell wall expansion and growth. This process involves a series of endogenous and highly regulated degradative activities (Figure 1), commonly known as autolysins [9]. However, PG degradation can also be performed by exogenous ‘predatory’ enzymes that target the bacterial envelope; some of which are common elements of the innate immune system of animals and plants [10]. Hence, bacteria employ various PG editing mechanisms to counter life-threatening lytic activities [9,11]. In this review, we highlight how bacterial cell walls adapt to environmental threats, emphasizing the remarkable plasticity of PG.

### Common peptidoglycan structural changes

Bacteria can alter the composition of their cell wall by synthesizing new cell wall components, modifying existing ones, or degrading others. All these modifications can influence the strength or flexibility of the cell wall, its susceptibility to antibiotics, or bacteria's ability to interact with host cells or the environment [12,13].

#### Peptide moiety

**Figure 2.**
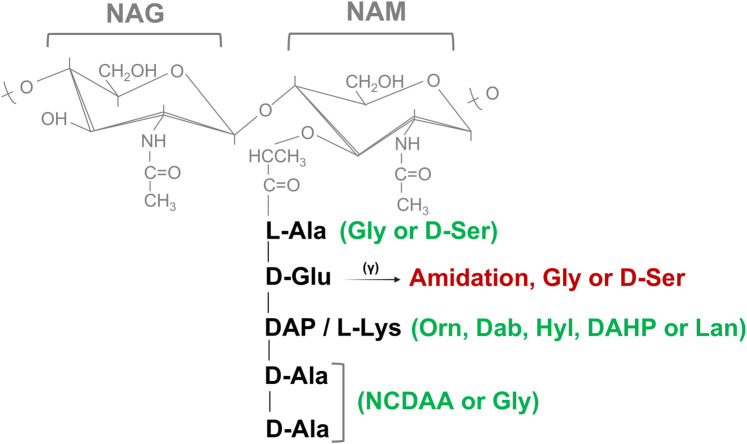
Structural substitutions and modifications in the peptide stem. Possible structural substitutions (in green) and modifications/links (in red) to the α-carboxylate of D-Glu are described at the peptide moiety in peptidoglycan. NAG, *N*-acetyl-glucosamine; NAM, *N*-acetyl muramic acid; L-Ala, L-Alanine; D-Glu-(γ), D-glutamic acid; DAB, 2,4-diaminobutyric acid; DAHP, 2,6-diamino-3-hydroxypimelic acid; Hyl, hydroxylysine; Lan, lanthionine; Orn, ornithine; DAP, meso-diaminopimelic acid; L-Lys, L-lysine; D-Ala, D-Alanine; D-Ser, D-Serine; Gly, glycine; NCDAA, non-canonical D-amino acids.

Lysine is frequently the third amino acid of the peptide moieties of PG in the majority of Gram-positive bacteria (Lys-type PG), whereas mDAP residue is present in this position in Gram-negative and also in rod-shaped Gram-positive bacteria such as *Bacillus* or *Clostridium* spp. [2]. However, variations in the third amino acid position inside the pentapeptide side chain such as 2,4-diaminobutyric acid (DAB), 2,6-diamino-3-hydroxypimelic acid (DAHP), hydroxylysine (Hyl), lanthionine (Lan), or ornithine (Orn) has been also described [2,11,14]. In addition, various strains of *Lactobacillus*, *Micrococcus*, *Staphylococcus*, *Streptococcus* and *Corynebacterium* spp. amidate the α-carboxylate of D-Glu and occasionally bind it to additional amino acids such as Glycine or D-Serine (D-Ser) [2]. Most bacteria can also edit the chemical composition of the peptide termini of the PG (Figure 2) by incorporating non-canonical D-amino acids (NCDAA, i.e. D-amino acids other than D-Ala or D-Glu) [11,15–17]. PG editing by NCDAA is controlled in the pathogen *Vibrio cholerae* by the general stress sigma factor RpoS and involves DD- or LD-transpeptidase activities, depending whether the substitution occurs in the fifth or fourth position, respectively [16]. Components other than amino acid such as D-Lactate (D-Lac) have been also reported to replace D-Ala at fifth position of the peptide moiety [2,14].

While it is not fully understood the biological purpose for some of these edits, some confer resistance to certain cell wall acting antibiotics such D-Ser or D-Lac to vancomycin in Gram-positive bacteria [17,18] while others, such as the incorporation of D-Met in *V. cholerae*, down-regulates PG synthesis during the transition from exponential to stationary growth phase [15,19]. Instead, *Acinetobacter baumannii* decorates its PG with the NCDAA D-Lys to gain protection against PG-targeting type VI secretion system effectors from bacterial competitors [20]. Although no systematic studies have yet investigated the effects of NCDAA-editing in PG on immune modulation, it is suggested that these substitutions may help bacteria evade inflammation by avoiding recognition by human NOD-like receptors [21]. This evasion could facilitate bacterial spread during infection.

#### Cross-linking

Bacteria can modulate the degree of cross-linking in their PG. In general, cross-linking homeostasis depends on multiple proteins (Figure 1), including PG synthetic enzymes such as DD and LD-transpeptidases as main catalyzers (e.g. high molecular mass PBPs or LD-transpeptidases), but also PG hydrolases such as carboxypeptidases (e.g. low molecular mass PBPs or LD-carboxypeptidases), endopeptidases and amidases, which affect the availability of the substrate monomers or the turnover of the cross-linked PG [12]. Alternatively, some Gram-positive species cross-link their PG using bridges of one or more amino acids. Modifications in the length and number of these bridges (e.g. penta-glycine bridges in *Staphylococcus aureus*), can affect the rigidity and flexibility of their cell wall [16]. Consequently, mutations or changes in the expression of the genes encoding these enzymes often lead to alterations in the cross-linking [11,12,17].

To preserve cell wall integrity under certain environmental conditions, some bacteria can transition their cross-linking pattern from DD-type to LD-type using envelope stress response systems [22,23]. In a study, Δ*ldcV* mutants in *V. cholerae*, which are incapable of processing their recycled PG peptides, accumulate tetrapeptide precursors within their cytoplasm. This accumulation leads to a significant reduction in DD-cross-links as PBPs cannot utilize tetrapeptides in DD-transpeptidation reactions. To compensate for this deficiency, increasing the expression of LdtA — the primary LD-transpeptidase in *V. cholerae* — enhances the fitness of the Δ*ldcV* mutant. Since *V. cholerae* LdtA is controlled by the general stress sigma factor RpoS [19] these findings highlight the crucial cross-talk between DD- and LD-cross-links in maintaining PG structural homeostasis under stress [24]. In this line, three previously uncharacterized LD-transpeptidases in *Pseudomonas aeruginosa* contribute to resistance against EDTA's bactericidal effects and facilitate the adaptation of cell envelope polymers to biofilm conditions [25].

In Mycobacteria, LD-transpeptidases are crucial for maintaining rod shape, particularly in aging cell wall regions resulting from polar growth. When LD-transpeptidases are absent, PBPs take on a more significant role in cross-linking PG. This vulnerability can be exploited in treating *Mycobacterium tuberculosis* by using a combination of drugs that target both DD- and LD-transpeptidases [26]. These interplay between distinct cross-linking mechanisms in maintaining cell wall integrity has also recently been demonstrated in *Gluconobacter oxydans* [7]. This acetic-acid bacterium encodes a novel type of LD-transpeptidase, conserved in many α- and β-proteobacteria, which generates L-Ala-mDAP (1,3-cross-links) instead of the canonical mDAP-mDAP (3,3-cross-links). The turnover of DD-cross-links supplies the necessary substrate for the β-lactam-insensitive LD_1,3_-transpeptidation. Consequently, *G. oxydans* lacking LD-cross-links is significantly more sensitive to ampicillin compared with the wild-type strain [7].

Recent research has demonstrated that LD-cross-links inhibit the activity of lytic transglycosylases (LTs), a type of autolysin responsible for breaking down the PG glycan chains. This regulation not only controls the release of immunogenic PG fragments but also provides resistance against predatory LTs from both bacterial and viral sources [27]. These findings contribute to our understanding of the physiological role of LD-cross-linking in PG homeostasis, showing how bacteria can enhance their resilience against environmental threats, including phage attacks, through a single structural modification of PG. Although it remains to be studied whether LD_1,3_-cross-links exhibit similar LT-inhibitory properties, the fact that LD-transpeptidases are generally insensitive to β-lactam antibiotics and their products can down-regulate LT activities suggests that this cross-linking mode safeguards the balance between PG synthesis and turnover in various ways.

#### Glycan moiety

Several modifications of the NAG and NAM sugars have been reported (Table 1) with implications in permeability and resistance of the cell wall to environmental stressors [13]. The main modifications described are:

**Table 1.** Chemical modifications of peptidoglycan disaccharides

	Unmodified disaccharide	*N*-deacetylation	*O*-acetylation	*N*-glycolylation	1,6-Anhydro-NAM residues	δ-Lactam residues
Structure variation in disaccharide	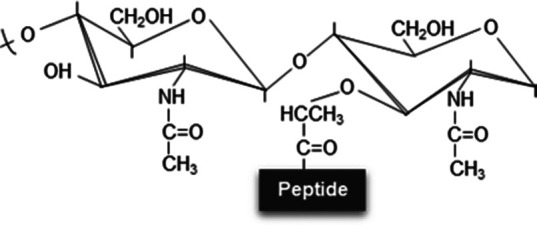	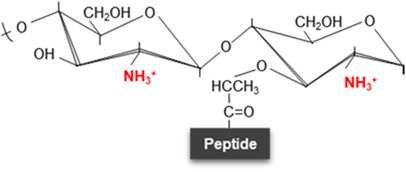	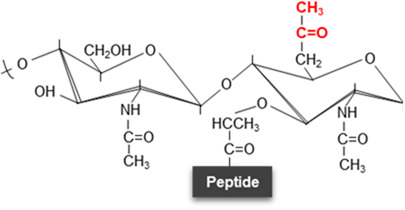	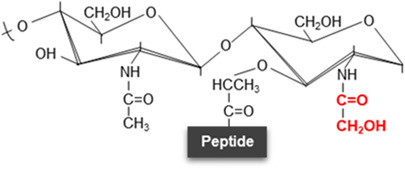	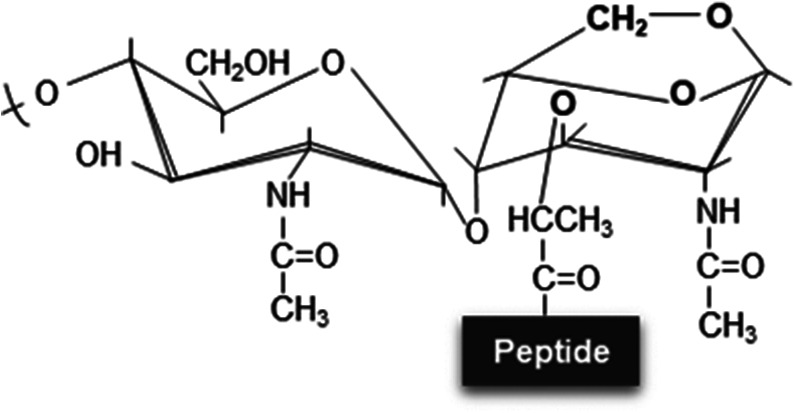	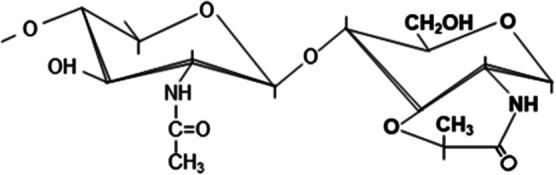
Modified sugars	-	NAG, NAM or both	NAM (with the exception of *L. plantarum* which shows *O*-acetylation at NAG) [46]	NAM	NAM	NAM
Inducer	-	Innate immune system (mucus, macrophages, neutrophils, etc.), phages or peptides with lytic activity	Environmental stressors such as antimicrobial compounds	Unclear, allegedly antibiotics that interfere with PG synthesis and environmental stressors	β-Lactam exposure (decreased transpeptidase activity)	Sporulation and adverse conditions
Chemical modification	-	Cleavage between the acetyl group and nitrogen atom	Addition of an acetyl group to the C6 hydroxyl group	Glycolyl residue (instead of acetate) at the two-amino group	1,6-Anhydro-NAM ring	Cyclic amide with six-atom rings
Responsible enzyme	-	*N*-deacetylases (e.g. PgdA or PdaA)	*O*-acetyltransferases (e.g. OatA or PatA/B)	UDP-*N*-acetylmuramic acid hydroxylases (e.g. NamH)	Lytic transglycosylases (LTs)	Muramoyl-l-alanine amidases (e.g. CwlD)
Effect	-	Moderate resistance to lysozyme and other lytic enzymes	Adaptation, resistance to lytic enzymes or control over autolysins	Alleged immune system evasion, β-lactam resistance and cell-wall stability	Overexpression of chromosomally encoded β-lactamases (β-lactam resistance)	Stability, heat resistance, DNA protection against radiations and antimicrobial resistance of the spore structure
References	-	[16,17,28,30,31,105]	[17,40,41,106–108]	[17,47,48,109]	[6,49,51–53,89]	[17,58,110,111]

Summary of the different chemical modifications of the disaccharide structure of PG indicating which sugars are affected, as well as the cause inducing the change, the enzymes responsible and their biological benefit.

##### *N*-deacetylation

This modification is catalyzed by PG deacetylases that break the bond between an acetyl group and a nitrogen atom of the sugar producing glucosamine from NAG or muramic acid from NAM (Table 1). It was first noted in the 1970s and 1980s in lysozyme resistant strains of several *Bacillus* spp. strains, and later also in *Lactobacillus fermentum*, *Listeria monocytogenes*, *Micrococcus lysodeikticus*, *Streptococcus pneumoniae* and *Helicobacter pylori* [16,17]*.* Deacetylated PG strands are poor substrates for lysozyme or muramidase activities, as these enzymes recognize the acetyl groups of the PG to cleave between NAM and NAG (Figure 1, Table 1) [17,28]. Hence, this alteration frequently takes place as a defensive mechanism against lysozyme activity originating from various sources, including phages, bacteria, fungi [29] and even mammals. Therefore, this PG modification is particularly crucial for pathogenic bacteria to evade innate immune system factors with PG-degrading activity, such as lysozyme [30,31].

The enzyme PgdA catalyzes NAG deacetylation in Gram-positive bacteria, influencing cell wall integrity and the evasion of the host immune system. Mutants lacking PgdA exhibit increased sensitivity to lysozyme and reduced virulence [29,31]. Additionally, NAG deacetylation plays a role in bacterial predation [32], protection against autolysins [33] and pathogenesis [34]. The activity of PgdA is regulated by cell division proteins, and its relevance extends to oxidative stress and infection [35].

The *N*-deacetylation of NAM by enzymes like PdaA has been associated with anchoring of the poly-γ-D-Glu capsule in *Bacillus anthracis* [36]. PdaA-like enzymes may help non-sporulating bacteria as *Rhizobium leguminosarum* evade immunity by facilitating muramyl dipeptide (MDP) binding to NOD2, triggering immune responses [29]. A recent study identified an *N*-deacetylase in the plant pathogen *Agrobacterium tumefaciens* that specifically removes acetyl groups from 1,6-anhydro-NAM chain termini. This discovery explains why canonical anhydromuropeptides were previously undetected in the PG of this bacterium. Additionally, it suggests that this modification could enable bacteria to regulate LT activity, thereby controlling chain length [37]. Although several studies indicate that *N*-deacetylases could potentially be used as drug target, in certain pathogens such as *Clostridium difficile*, *N*-deacetylation does not seem to affect colonization or virulence [38]. One recent study in *C. difficile* highlights the involvement of the *N*-deacetylase PdaA1 in cleaving the acetyl moiety of NAM, catalyzing a unique zinc-dependent transamidation/transpeptidation reaction [39].

##### *O*-Acetylation

*O*-acetylation involves adding an acetyl group to the C6 hydroxyl group of NAM (Table 1) [17]. This modification typically occurs in pathogenic Gram-positive and Gram-negative species, including *S. aureus*, *Bacillus subtilis*, *Enterococcus faecalis*, *Clostridium botulinum*, *Streptococcus pyogenes*, *Neisseria gonorrhoeae*, *Proteus mirabilis*, *H. pylori*, and *Moraxella catarrhalis*. However, it is absent in other bacteria such as *Escherichia coli, P. aeruginosa, B. anthracis*, and non-pathogenic staphylococci. The enzymes responsible for *O*-acetylation are usually OatA in Gram-positives and PatA/B in Gram-negatives [40].

Several studies indicate that *O*-acetylation regulates endogenous autolysins involved in PG maturation and cell division [40,41]. Additionally, *O*-acetylation provides protection against exogenous PG-degrading enzymes like lysozyme by blocking access to NAM residues in the PG backbone [41]. In *S. aureus*, *O*-acetylation helps shield the bacterium from host lysozymes and neutrophils, potentially contributing to disease by hindering innate immunity during the recognition stage [42,43]. Consistently, OatA mutations in *S. aureus* are associated with virulence attenuation, sometimes alongside other advantageous mutations related to antimicrobial resistance [44].

A comprehensive study of vancomycin-resistant *E. faecalis* (VRE) revealed that growth in the presence of vancomycin leads to reduced PG cross-linking, increased carboxypeptidase activities, elevated *N*-deacetylation, and enhanced *O*-acetylation. Notably, *O*-acetylation occurs preferentially in regions with decreased PG cross-linking, specifically on units terminating in D-Ala-D-Lac. These regions act as markers to resist modifications and prevent cell wall degradation, contributing to lysozyme resistance and increased virulence in VRE exposed to vancomycin [45]. Interestingly, *O*-acetylation of NAG has been exceptionally found only in *Lactobacillus plantarum*, suggesting its rarity in bacteria. The dedicated *O*-acetyltransferase responsible for this process is called OatB. However, unlike lysozyme resistance, *O*-acetylation of NAG inhibits the activity of the major autolysin Acm2 in *L. plantarum* [46]. These findings raise intriguing questions about the evolutionary significance of this modification, its biological role across different species, and its potential as a target for new antimicrobial therapies.

##### *N*-glycolylation

*N*-Glycolylation of NAM involves adding a glycolyl residue to replace acetate at the 2-amino group (Table 1). This modification is primarily found in Actinomycetales, including *Mycobacterium* species [17]. The degree of *N*-glycolylation varies among species and in response to different antibiotics. The enzyme NamH catalyzes this process, and its deletion in *Mycobacterium smegmatis* increases sensitivity to β-lactam antibiotics and lysozyme. Blocking PG synthesis, which leads to the accumulation of cell wall precursors, increases *N*-glycolylation. This indicates that NamH acts on the UDP precursor pool [29]. Although *N*-glycolylation-deficient mutants do not exhibit alterations in cell shape or growth [47,48], *N*-glycolylation is believed to contribute to the structural stability of the cell wall architecture [17,48]. While the specific molecular and structural changes that enhance cell wall stability following NAM *N*-glycolylation are not fully understood, this increased stability likely helps resist β-lactams and lysozyme. Consequently, *namH* mutants are more sensitive to these compounds. Furthermore, *N*-glycolylated NAM enhances NOD2 recognition, though its role in *M. tuberculosis* infection appears limited [47].

##### 1,6-Anhydro-NAM

The presence of 1,6-anhydro-NAM residues indicates the end of a glycan chain (Figure 1, Table 1). When 1,6-anhydro-NAM increase, the PG chains become shorter in average and the sacculi structure gets more flexible [6]. During cell growth in Gram-negatives, regular turnover of PG by LTs and endopeptidases release these PG fragments outside the cell. 1,6-anhydro-NAM residues are common in Gram-negatives and occurs infrequently in Gram-positives, typically in low proportions due to their less dynamic cell wall turnover [17,49].

Many Gram-negative bacteria re-internalize these 1,6-anhydro-NAM fragments into their cytoplasm through permeases (e.g. AmpG) or ABC transporters [9,50]. Recycling of these muropeptides is further facilitated by degrading activities such as glucosaminidases (e.g., NagZ) and amidases (e.g. AmpD). Upon inhibition of PG synthesis by β-lactams, uncontrolled autolytic enzymes lead to supra-accumulation of 1,6-anhydromuropeptides that can induce chromosomally encoded β-lactamases as AmpC in some Gram-negative bacteria such as in *Citrobacter freundii*, *Enterobacter* spp. or *P. aeruginosa* [51–53]. Gram-positive bacteria, where muropeptide transporters are almost universally absent, rely instead on peptide and sugar transporters to recycle the PG fragments released during PG turnover [54,55].

##### δ-Lactam NAM

This cyclic amide with six-atom rings (Table 1) has been found mainly in the PG of bacterial spores from *Bacillus* spp. or *Clostridium* spp. [17]*.* The production of spores allows the bacteria to remain latent for extended periods, potentially years or even decades, potentially by making them resistant to heat, UV radiation and impermeable to antibiotics [56]. In the context of the intestinal microbiota, spore germination commonly occurs after antibiotic treatment, contributing to the dysbiosis [57]. The production of δ-lactam residues involves a series of enzymatic steps. First, muramoyl-l-alanine amidases (such as CwlD in *B. subtilis* and *C. difficile*) cleave the peptide bound to muramic acid. Next, the resulting product undergoes *N*-deacetylation. Finally, a transpeptidase creates the muramic δ-lactam ring. In *B. subtilis* mutants lacking either of these enzymes, spores have PG completely depleted in muramic δ-lactam content. Consequently, the germination process is interrupted due to the absence of cortical hydrolysis [38,58].

Despite extensive knowledge about PG modifications and their roles in resistance and immune evasion, many questions remain unanswered. We still lack a full understanding of the environmental conditions and regulatory factors driving specific structural changes in the bacterial cell wall, as well as the evolutionary mechanisms behind strain-specific variability. Additionally, the impact of these modifications on bacterial survival, resistance, and adaptation under various stress conditions is not yet fully clear, underscoring the need for more in-depth research in this area.

### Cell wall modifications during host-pathogen interaction

Host NOD-like receptors detect PG fragments to subsequently activate inflammatory responses via NF-κB [59]. NOD1 specifically recognizes structures containing mDAP, while NOD2 exclusively recognizes MDP fragments [60]. To evade host recognition, *M. tuberculosis* increases amidation and glycosylation of its PG inside phagocytic cells to down-regulate NOD2 signalling [61–63]. Similarly, Gly substitutions for L-Ala have also been detected in PG isolates of *Mycobacterium leprae* resulting in reduced NOD2 receptor activation [64]. The PG of intracellular non-proliferating *S. enterica* serovar Typhimurium infecting fibroblasts increases the degree of LD-type cross-links and replaces the terminal D-Ala of its peptide chain with the amino alcohol alaninol, a modification that appears instrumental to evade immune recognition [65,66]. Additionally, *S. enterica serovar* Typhimurium infecting fibroblasts and dendritic cells induce the DL-endopeptidase EcgA which cleaves between D-Glu and mDAP in the PG, thereby making the resulting MDP fragments not recognizable by the NOD1 receptor and hence, contributing to its infection and persistence [67,68].

In addition, *N*-deacetylase enzymatic activity might also assist some bacteria in avoiding the innate immune system. A study involving synthetic structural analogues of MDP revealed that the presence of the acetyl group in NAM is essential for MDP binding to the NOD2 receptor and subsequent signaling [69]. The innate immune PG recognition proteins (PGLYRPs) types 1, 3, and 4, commonly found in mammals, have been shown to elicit bactericidal effects by activating the two-component CpxA-CpxR system upon binding to Gram-negative surfaces [70,71]. Several studies demonstrated that activation of CpxA-CpxR leads to increased mDAP-mDAP cross-linking (up-regulating specific LD-transpeptidases) and hence, enhanced resistance to β-lactams [22,23,71]. However, excessive CpxAR activation by PGLYRPs cause aberrant bacterial morphology and growth defects ultimately resulting in heightened susceptibility to β-lactams [23].

Gram-negative bacteria employ various strategies to evade host immune detection, including surface structures like lipopolysaccharides (LPS) and capsular serotypes, as well as internal PG modifications. LPS and capsular polysaccharides undergo numerous modifications that help them evade immune recognition without compromising bacterial viability [72,73]. In contrast, PG modifications, though less exposed, are crucial for avoiding detection by intracellular immune sensors such as NOD1 and NOD2. The exact impact of these sugar and peptide modifications on NOD1 and NOD2 signaling, and consequently on bacterial resistance and immune evasion, remains poorly understood.

### Enhancing resilience in challenging environments: cell wall autolysis strategies

Autolysins are tightly controlled PG degrading enzymes that play crucial roles in cell division, PG turnover and the correct assembly of envelope-anchored structures such as the flagellum or toxin secretion systems [5,74]. The genetic basis of autolysis and its regulation largely depend on the bacterial species and the specific stress conditions. Expression of these enzymes is often controlled by cell wall stress responsive sigma factors [75,76] and two-component systems such as WalKR [75], LytSR, CiaRH [77,78] and VraSR (vancomycin resistance associated) [79] in Gram-positives or the Cpx and Rcs systems in Gram-negatives [23,80]. Outer membrane lipoproteins, such as NlpI, have been also shown to regulate autolysins’ activity in Gram-negative bacteria [81].

**Figure 3.**
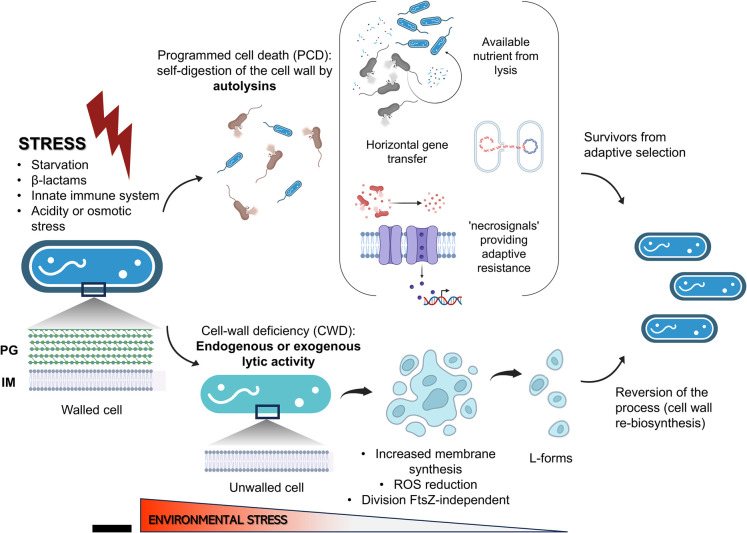
Bacterial adaptation to environmental stress. Schematic diagram explaining how bacteria adapt to environmental stresses: In the presence of environmental stress, bacteria have several adaptive strategies such as programmed cell death (PCD) in certain cells of the population to provide nutrients and recyclable metabolic remnants for the survivors. They also provide genetic material and post-lysis signals capable of modifying the expression of resistance determinants. Another option is the overexpression of autolysins to produce protoplasts or unwalled cells, which are unable to proliferate due to oxidative damage brought on by the buildup of reactive oxygen species (ROS), from aerobic respiration products. L-forms are created when mutations that lower ROS levels work in conjunction with enhanced membrane production to allow development without the cell wall. L-forms can multiply without *ftsZ* (key gene in bacterial cell division) and give rise to asymmetric progeny with different numbers of chromosomes. It should be noted that a monoderm bacteria is depicted in this design. PG, peptidoglycan; IM, inner membrane.

Environmental stresses such as nutrient deprivation, exposure to β-lactams or changes in temperature, pH or osmolarity, can trigger these activities [82,83]. For instance, LTs from bacterial and viral origin can be inhibited by changes in the degree of LD-cross-linking in the PG, which is controlled by stress inputs. For example, LdtA is regulated by RpoS in *V. cholerae* [19], and LdtD by CprX in *E. coli* [22]. While this mechanism of autolysin control likely supports bacterial survival at the level of an individual, such as protecting a cell from being lysed by phage or bacterial type VI-injected LTs; in other cases, autolysins initiate emergency processes, including programmed cell death (PCD), in which a subpopulation dies to benefit the community by providing nutrients (Figure 3) [83,84]. PCD indirectly contributes to antibiotic resistance through ‘necrosignals’ released after lysis. For example, release of the periplasmic protein AcrA can be sensed by the TolC membrane protein of surviving cells. This interaction stimulates the induction of efflux pumps thereby providing adaptive resistance to resilient subpopulations (Figure 3) [85]. Other benefits resulting from PCD lysis include release of DNA and signaling molecules important in horizontal gene transfer [86,87] and in the modulation of gene expression in biofilms [88]. However, autolysis can also be detrimental to bacterial survival if is not well regulated, therefore, the regulation of autolysis is a critical aspect of bacterial survival and adaptation [17,89–91]. Further research will elucidate the precise mechanisms by which stress-responsive systems regulate autolysins and how PCD and necrosignaling affect antibiotic resistance and gene transfer.

### Peptidoglycan depletion as a survival mechanism

Although a few bacteria such as Mycoplasmas lack PG [92] many other species can reversibly transition into cell-wall deficient (CWD) cells, known as L-forms [84,93] to adapt to certain environments. For this process to take place, the role of autolysins or exogenous lytic enzymes is strictly necessary (Figure 3).

Under certain stresses, PG synthesis may halt, while autolysins continue to act. Alternatively, exogenous lytic enzymes, such as host lysozyme, can disrupt the balance between synthesis and turnover. In either scenario, bacteria often transition to spherical CWD forms [94]. These CWD forms are characterized by inhibited PG synthesis (for example, *B. subtilis* mutants with mutations in the *ispA* gene, which is essential for PG synthesis) and an excess of cell membrane production [95]. This membrane overproduction has been associated with the ability of these cells to proliferate via an ancestral *ftsZ*-independent method that produces asymmetric progeny (Figure 3) with the possibility of having several copies of the genetic material. How these stresses mechanistically induce to the unwalled state are unknown [84,93,96,97].

While a CWD lifestyle may render cells more fragile and susceptible to lysis due to abrupt osmotic changes, paradoxically, the absence of PG provides unique survival opportunities that a mature PG cannot offer. Some of these advantages include resistance to cell wall-acting agents like β-lactam antibiotics and host lytic enzymes (such as lysozyme) [98]. Additionally, CWD cells are not recognized by the host immune system due to the absence of Pathogen-Associated Molecular Patterns recognized by pattern recognition receptors [99,100]. The reversibility of the CWD process allows cells to reinfect and adapt [94,101]. CWD bacteria are present in the urine of most older patients suffering from recurrent urinary tract infections (rUTI). These bacteria switch between walled- and L-forms in response to antibiotics [102], suggesting they play a significant role in the persistence and recurrence of infections in these patients. Furthermore, studies have shown that CWD protoplasts have a greater capacity for horizontal gene transfer [87]. Lastly, CWD cells exhibit increased resistance to phage infection because the absence of PG prevents proper binding of receptor-binding proteins on phages, reducing susceptibility to phage invasion [103,104]. While the benefits of PG synthesis depletion in antibiotic resistance and immune system evasion are evident, the exact mechanisms by which stress triggers the transition to CWD forms, and the roles of autolysins or exogenous lytic enzymes in this process, remain largely unclear.

### Concluding remarks: deciphering the structural modifications of peptidoglycan to delineate new therapeutic strategies

Bacteria dynamically remodel their PG to adapt to various environmental challenges, including those posed by cell wall-acting antibiotics. Additionally, PG remodelling is commonly employed during host-pathogen interactions, allowing bacteria to establish infections by evading host defences. Understanding these adaptive strategies enriches our knowledge and inspires novel therapeutic approaches. By targeting bacterial cell wall vulnerabilities, we navigate the delicate balance between survival and host defence mechanisms.

## Perspectives

- Bacteria adapt their cell walls through structural modifications, such as peptide and glycan alterations, and by transitioning to CWD forms to survive environmental stresses and evade immune responses.
- Future research should focus on unravelling the mechanisms behind these adaptations and their biological consequences for the bacteria and the host.
- Exploiting PG adaptive changes will offer novel cell wall vulnerabilities and therapeutic opportunities.

## Abbreviations

CWD: cell-wall deficient
LPS: lipopolysaccharides
LT: lytic transglycosylase
MDP: muramyl dipeptide
mDAP: meso-diaminopimelic
NAG: *N*-acetylglucosamine
NAM: *N*-acetylmuramic
NCDAA: non-canonical D-amino acid
PBP: penicillin-binding proteins
PCD: programmed cell death
PG: peptidoglycan
VRE: vancomycin-resistant *E. faecalis*
